# “*Woman’s Hour* or Mother’s Hour”: postnatal depression narratives, treatment and reception on BBC radio, 1946–1985

**DOI:** 10.1080/14680777.2025.2468897

**Published:** 2025-03-05

**Authors:** Fabiola Creed

**Affiliations:** School of Social & Political Sciences, University of Glasgow, Glasgow, UK

**Keywords:** BBC radio, *Woman’s Hour*, motherhood, women’s narratives, maternal mental illness

## Abstract

Launched in 1946, *Woman’s Hour* became one of Britain’s first women-organised radio programmes, predominantly targeting mothers. In 1960, *Woman’s Hour* transmitted the first mass media broadcast on “childbirth depression” in Britain; however, such discussions only became standard on *Woman’s Hour* in the twenty-first century. This article explores why the BBC and *Woman’s Hour* developed an interest in maternal mental health. It then evaluates how *Woman’s Hour* approached the originally “taboo” topic by comparing the narratives (doctors, women and “husbands”), treatments (medical, pharmacological, and hormonal), and reception (absent to varied) pertaining to three postnatal depression features produced in 1960, 1974, and 1985. Most researchers of motherhood explore print press and increasingly television sources, yet radio remains overlooked. This article maps how this medium navigated and reflected key changes in Britain’s gendered workplace and family dynamics. The article builds on and contributes to the history of changing attitudes to motherhood and maternal mental illness, radio as a form of health communication, the influence of women’s voices, and medical professionals’ expanding responsibilities in post-World War Two Britain. This will appeal to scholars from many disciplines, including history, gender, culture, and feminist media studies.

## Introduction

In 1922, the BBC transmitted daily radio broadcasts for the first time in Britain. During World War 2, BBC radio became the nation’s mainstream media as people relied on the broadcaster for war updates (Paul Rixon 2018). In 1946, the BBC launched *Woman’s Hour*, one of the first radio programmes organised by mostly female staff for other women listening at home, marking a mass media milestone for women (Kate Murphy 2022, 213). Almost eighty years later, *Woman’s Hour* is still broadcasting. It remains one of Britain’s most enduring and widely known radio programmes (Eduqas 2021–2022). Therefore, a chronological exploration of how a topic has been addressed on *Woman’s Hour* offers an invaluable method for researchers to study key changes in socio-cultural attitudes, mass media, and women’s health advice in Britain.

This article offers one such example by illustrating how *Woman’s Hour*’s features on postnatal depression shifted from being absent between 1946 and 1959 to featuring every year in the twenty-first century, even though listeners requested such discussions long before. In 1947, for instance, the veteran BBC Talks Producer Isa Benzie wrote to Eileen Molony, *Woman’s Hour*’s editor, with requests from listeners’ letters. Although the original letters are lost, Benzie had compiled a list of listeners’ questions, showing “the … things which listeners consistently need help and … are deeply interested in.” One of these questions included, “Could you give a short talk on depression” in relation to “motherhood and children” (Isa Benzie 1947, 5–7). As historian Hilary Marland (2004) argues, “puerperal melancholia” existed long before WW2. Yet, the vague request reflected most women’s lack of awareness and understanding of maternal mental illness in the 1940s (Teri Chettiar 2023, 80–105; Sarah Crook 2016, 10). Nonetheless, the first report on “childbirth depression” aired in 1960 because the BBC as a whole remained anxious when broadcasting “taboo” mental health topics in the 1940s and 1950s (Vicky Long 2004, 296–348). Moreover, the BBC perhaps perceived a mental illness that contravened women’s joys of “natural” and “central” motherhood as more taboo than others (Chettiar 2023, 80–1).

An exploration of postnatal depression on *Woman’s Hour* in post-war Britain will illustrate how the media, medical experts, and everyday people gradually discussed maternal mental illness, and general mental health, more openly. Historians have already studied how the print press and television programmes raised people’s awareness and understanding of maternal mental illness. Yet, they have overlooked women’s narratives or lack thereof on radio, and how radio differed from other media (Crook 2016; Fabiola Creed 2024). Building on Jane Arthurs’ and Usha Zacharias’s (2007, 338–41) work, this article also offers a method for researchers to explore how *Woman’s Hour* radio approached social issues and taboo topics in contemporary Britain. Finally, the unique focus on *Woman’s Hour*’s postnatal depression features will build on the history of changing attitudes to motherhood and maternal mental illness, radio as a form of health communication, women’s voices and agency, and the expanding responsibilities of medical professionals in post-war Britain.

The article begins by exploring why the BBC and *Woman’s Hour* developed an interest in mothers’ mental health, particularly postnatal depression. It then evaluates the differences in how *Woman’s Hour* approached the topic by comparing three postnatal depression features produced in 1960, 1974, and 1985. These three features were the only ones referenced in all radio guides and the BBC Written Archive (BAC). In the twentieth century, many BBC radio broadcasts were not recorded or were later wiped. In contrast, most twenty-first-century episodes are stored online, making it easier to map recent changes. Drawing on the first three archived features, this article will map how the twentiethcentury broadcasts both reflected and contributed to the de-stigmatisation of maternal mental illness, shifting radio testimonies from taboo to mainstream in Britain.

To study these changes, I analysed sources from the BBC Written Archives (*Woman’s Hour* transcripts), the autobiographies of *Woman’s Hour* employees, and the publications of the interviewed medics. *Woman’s Hour*’s transcripts reveal interviewees’ and, via their letters, listeners’ experiences and opinions. From 1946 to the 1990s, listeners sent hundreds of letters every week.

## The BBC, *Woman’s Hour* and maternal health

Many historians, including Rodney Lowe (1998, 80), agree that Britain’s welfare state formally emerged when the public funded National Health Service (NHS) came into operation in 1948; however, historians also maintain that the government launched a series of welfare reforms to advance health decades long before (Roger E Backhouse and Tamotsu Nishizawa 2010, 1–22). When John Reith launched the BBC in 1922 as a public service to “educate, inform and entertain,” it was heavily influenced by the government’s aim to improve people’s welfare. Consequently, sociologist Tom Mills (2020) argues that the BBC supported the government on most matters while pressurising producers and presenters to efface their opinions in favour of “editorial balance.”

Before the BBC launched *Woman’s Hour*, the population losses of two world wars, the Great Depression, and falling birth rates in the 1930s and 1940s prompted governmental concerns about Britain’s weakening nation and depopulation (Angela Davis 2014, 257–66). Historian Rebecca Jennings (2013, 78) argues that children became a national resource, and their health—alongside their mothers—became the welfare state’s focal point. To encourage a population of healthy bodies, British institutions, including the BBC, focused on reducing maternal and infant mortality rates and improving the physical wellbeing of primarily white “British” mothers and babies (Wendy Webster 1998). As historian Chettiar (2023, 80–1) illustrates, the BBC greatly supported women’s “natural” and “central” roles as mothers. Consequently, if mothers experienced a mental breakdown, it contravened idealised motherhood. This resulted in guilt from and stigma towards “bad” mothers, and tabooed maternal mental illness.

The BBC launched *Woman’s Hour* (October 1946) weeks before the NHS Act (November 1946). Moreover, Britain’s new state practice of interventionism, evident in BBC media, emerged as “welfare support.” Britain’s government assumed a paternalistic right to intervene in people’s domestic affairs by providing health care, education, family allowances, and support for children—topics which *Woman’s Hour* dutifully covered (Murphy 2022, 210). The partnership between the BBC and NHS strengthened government, medical and media authority to “educate” women on improving family health (Anne Karpf 1988). By the 1950s, the BBC, via their “Home Service” channel, produced several “domestic” radio programmes, where BBC presenters and medics—mostly middle-class white men—offered health advice to improve mothers’ and babies’ physical health (Michal Shapiro 2013, 116–9). Following the decline in maternal and infant mortality by the 1950s, Britain’s medical and media institutions increasingly focused on mothers’ postnatal mental well-being (Katharina Dalton 1980, 7–8). Moreover, as historian Vicky Long (2014, 197–9) concludes, both the BBC and the British Medical Association (BMA) accepted mental illness programmes in the 1950s following new trends; medical bodies wanted favourable publicity, the BBC expanded their health programmes, and “new” treatments in mental healthcare were deemed “effective.” This perhaps explains why *Woman’s Hour* was one of the first radio programmes to broadcast a one-of-a-kind report on “childbirth depression” in 1960.

When the BBC started broadcasting in 1922, it experimented with a poorly received and, therefore, short-lived version of *Women’s Hour* (1923–1925) (Kate Murphy 2020). In 1946, *Woman’s Hour* was re-launched by two men, Norman Collins and Alan Ivimey. Janet Quigley, however, is credited for initiating the programme because of her expertise in developing “woman talks” since the 1930s (Murphy 2022, 209). Quigley used radio to assist Britain’s public policy, including its Ministry of Health’s agenda to improve “parentcraft”^1^; however, she wanted to remain free from direct government control. In the 1950s, Quigley pushed for taboo talks on childrearing and marital issues on *Woman’s Hour*, believing such topics should not be taboo. Consequently, Quigley took advantage of the *Woman’s Hour* policy of bringing “hush-hush” topics into the open (Paul Donovan 2004). This reflects how radio, through programmes like *Woman’s Hour*, originally broke new ground by diversifying audiences and content. This ceased when television took over radio as the dominant medium. However, it must be noted that *Woman’s Hour* producers, presenters, guests and listeners were predominantly white and almost exclusively focused on white motherhood in the twentieth century.

Many scholars have published on the history of *Woman’s Hour*. However, aside from Karpf (1988) and Justine Lloyd (2020), most focus on its earlier decades and women’s careers, not issues pertaining to motherhood (Maggie Andrews 2012; Macdonald Daly 2016, 131–41; Sally Feldman 2000; Murphy 2023, 2016; Jenni Murray 1996, 2006; Kristin Skoog 2017). Yet, the BAC holds invaluable sources on “mother and baby” talks from the 1950s when *Woman’s Hour* started collaborating with psychologists, such as paediatrician and psychoanalyst Dr Donald Winnicott (Robert Adès 2016, 233–8). Like Quigley, *Woman’s Hour* employees edited medics’ scripts, including Winnicott’s, to encourage empathy towards mothers and stop listeners from pathologising mother-child relations (Karpf 2014, 90–1). Moreover, *Woman’s Hour*’s growing focus on mother-child relationships likely paved the way for a long-awaited feature on “childbirth depression” in 1960, especially when listeners kept on “asking for it.”

## “Childbirth depression” on *Woman’s Hour* (May 1960)

On Tuesday, May 17 1960, between 2-3pm, *Woman’s Hour* transmitted a feature on “childbirth depression” for the first time (*Woman’s Hour* 1960), reaching up to four million people (Sue Macgregor 2003, 150). The broadcast on a taboo topic—and transmitted through the “Light Programme” channel (1946–1967)—marked a significant moment. In the 1950s, the “Light Programme” disseminated light entertainment for working- and lower-middle-class women who listened while undertaking domestic labour (Skoog 2017, 957–61). Consequently, a subject matter like “childbirth depression” was likely deemed too heavy, especially for men who also tuned in. Following the new introduction of radios in men’s workspaces in the 1950s, *Woman’s Hour* recognised that one-third of their listeners were men by 1960 (Andrews 2012, 119–20). Moreover, like Donald W Winnicott (1993, 2), most doctors in 1960 opposed “health education in mass form” because they feared it might attract people who were “morbidly” interested, and others might not fully listen (Karpf 1988). Nonetheless, in the 1950s, *Woman’s Hour*’s women producers and presenters—including Quigley, Benzie, Jean Burns, Jean Metcalfe and Joanna Scott-Moncrieff—urged their male managers and directors to broadcast listeners’ requests of more useful, albeit “hard-hitting,” health topics (Lloyd 2020, 101). Consequently, a male psychiatrist’s lecture on “childbirth depression” was perhaps a compromise.

When the feature aired, medical professionals had not yet reached a consensus on how pregnancy and birth affected women’s mental health. Additionally, many doctors lacked interest and could not agree on whether childbirth depression was different from general depression (M. E Martin 1958, 773–7). Nevertheless, women were clearly interested in talking about “childbirth depression” before medical experts, like Brice Pitt (1968), published on the condition in the late 1960s. The *Woman’s Hour* report was the first and longest (eight minutes) out of six other items on the hour-long programme. This generous length in radiophonic terms suggests that the producers wanted to get beyond the superficialities of the topic; *Woman’s Hour*’s producers took the features over six minutes more seriously (Daly 2016, 135).

Although *Woman’s Hour* broke traditional taboos by addressing the topic in 1960, they still had limitations. *Woman’s Hour*’s non-taboo features typically consisted of an interview between both a named women presenter and interviewee(s) who had relevant professional or lived experience (Sally Feldman 2007, 338–41). This approach helped maintain a familiar “shape” for each episode, including “appropriately feminine” voices (Olive Shapley 1996, 125). However, health issues were typically restricted to unnamed medical experts, usually men. In the 1950s, less than 25% of qualified medical doctors were women (Gianetta Rands 2018, 32–3) because they did not have the same education and career opportunities as men (Claire Hilton and Tom Stephenson 2019, 47–9). If there were comparable women experts, they were not invited to speak on *Woman’s Hour*. Consequently, Dr Russell Barton, a psychiatrist, scripted the childbirth depression lecture. The producer, Jean Burns, organised and likely edited the feature, and the experienced compère, Marjorie Anderson, introduced the topic but neither spoke during Barton’s lecture. *Woman’s Hour*’s scripting, editing and pre-recording of Barton’s “anonymous medical lecture” was typical in 1960, demonstrating how radio broadcasts on mental illness were relatively new and cautiously developed.

Barton—a young, ambitious, and outspoken medic (Diana Gittins 1998, 67)—was a suitable choice for such a taboo topic not yet addressed in mainstream media, including newspapers, magazines, radio or television. Barton was the newly appointed and last physician superintendent at Severalls Hospital, Colchester (1960–1971). He was passionate about providing humane treatment and care for mentally ill patients and improving facilities, especially NHS psychiatric hospitals Hilton 2018, 307–35). Barton was described as a “maverick” who spoke his mind when others found it “politic to keep their own counsel” (Henry Rollin 2018, 35–6).

When Barton spoke on *Woman’s Hour*, he had been exposed to and cared for mothers who had experienced maternal mental illness (Gittins 1998, 129–30). In 1953, Barton undertook his psychiatry training at Maudsley Hospital. In 1959, he published *Institutional Neurosis*, which explained how hospital regimes harmed patients, including mothers, and offered ways to prevent this maltreatment, such as giving patients greater autonomy. Barton challenged the status quo by developing humane patient-centred psychiatric care, which was perceived as “radical” and therefore received extreme support and opposition (Hilton 2018, 308, 326). As a well-spoken, educated, and charismatic leader (Hilton and Stephenson 2019, 60), Barton was likely chosen because of his “appropriate” “Queen’s English” accent and didactic mode of address, which *Woman’s Hour* producers and editors carefully considered (Long 2004, 318). Although he was anonymous to listeners, his authoritative persona likely lessened the risk of backlash from the BBC’s managers and listeners if his lecture was considered controversial.

## The lecture: definitions, causes and treatment

Barton knew most listeners were unaware of “puerperal depression.” His introductory lecture, therefore, explained variations of the condition, the causes, and past and present treatments. The lecture aimed to improve people’s understanding of maternal mental illness and encouraged sufferers to ask for help. He first explained that it was customary for new mothers to experience a “short spell of depression and exhaustion” or “silly doubts” after giving birth, and not knowing this might cause “unnecessary anxiety.” He disclosed how his patients believed they were going “mad” because of these negative feelings and were too afraid to tell their husbands or doctors.

The second section challenged the romanticisation of childbirth. Barton explained how it was normal for mothers to feel frightened by their “disgust” towards their child, deeming themselves “unnatural” for such thoughts. He, again, suggested sharing this with a “sympathetic friend, nurse or doctor” to lessen feelings of “guilt.” Barton then tried to dispel the myth of the “natural” mother, explaining how “love” required time and could follow feelings of “strangeness and dislike” towards their baby. Not everyone, he claimed, had maternal feelings. Although *Woman’s Hour* supported Barton’s lecture—otherwise, it would not have aired—listeners could not hear that an everlasting absence of “maternal feeling” was an option. *Woman’s Hour*’s production team scribbled in, “maternal feelings may not be immediately available for dealing with the baby.” The BBC devotedly upheld motherhood as “naturally central” for all women, reflecting Britain’s broader culture (Chettiar 2023, 81–2).

In the third section, Barton addressed the longer-lasting “intense” depression; however, he only mentioned the symptoms of “restless[ness], hopeless[ness] … and an awful sense of futility.” He did not mention that intense depression could lead to infanticide, perhaps reflecting restrictions on what could be aired. The best Barton could do was reassure listeners that “intense” depression was “extremely rare,” occurring in less than one in every thousand births.

Barton then admitted that no one really knew what caused such depression and recounted some theories. These included “glandular disturbances” in pregnancy and childbirth, and psychological, social, economic, and religious factors. Barton referenced a survey of 42 cases of “post-puerperal illness[es]” conducted by a Bristol psychiatrist, which revealed that patients did not blame their husbands; half, however, attempted suicide because of feelings of guilt towards their loved ones. Barton asserted that this was “unnecessary” because most patients recovered, and those who did not could be helped by treatment.

The final sections reflected Barton’s unusually positive opinion on “effective” pharmacological and medical treatments. He noted that “gland[ular] extract” treatment—the injecting of animal testes, ovaries, and other organs—was no longer used because of Electroconvulsive Therapy (ECT) and new pharmacological treatments. Barton explained how ECT produced the most “outstanding results;” however, an injection for sleep and relaxation was more typically given. Barton also praised new “synthesised” medications for curing depression, which “rival[ed]” ECT. From the 1940s to 1960s, psychiatrists liberally trialled new medical and pharmacological treatments on people suffering from psychiatric illnesses (Gazdag Gábor and Gabor S Ungvari 2009). In the 1940s, psychiatrists positively regarded and made ECT a standard treatment for extreme cases of puerperal depression—even when women suffered from convulsions, short-term to permanent memory loss or, although rare, died. This “treatment” also continued until the mid1980s (*Woman’s Hour* June 13 1985a).

Although Barton positively addressed these treatments in *Woman’s Hour*, he opposed their use in his book *Institutional Neurosis*. Barton believed sedatives produced “apathy,” and although ECT “quieten[ed]” people, it had “no place in the long-term treatment of patients” (Russell Barton 1959, 18–19). However, one of the BBC’s main objectives when broadcasting on mental health was to reassure people that treatment was successful. Therefore, Barton concluded his lecture with reassurance that childbirth depression could be cured by NHS doctors.

The typed script had an additional final section. However, this was crossed out and not transmitted to listeners. Barton had advised listening husbands and relations to offer women “extra kindness and understanding” after giving birth. He had instructed people to sympathetically listen and not tell depressed women to “cheer up” or “pull [themselves] together.” He also advised loved ones to speak to a ward sister or doctor if they were concerned. As this section was omitted, Barton could not suggest that the responsibility of a mother’s recovery be shared across the wider community; it remained the responsibility of the sufferer to ask for help. The crossed-out section was either deliberate or because the feature was already long. Either way, it suggests it was considered less important than the others.

Barton was clearly empathetic towards new mothers. His lecture was written to raise both awareness and support for suffering mothers. Barton, however, uncharacteristically supported medical and pharmacological treatment to appease the BBC, even though he personally challenged this approach. Moreover, Barton had to reassure husbands that women did not blame them for their mental illness, and he could not encourage husbands to help with their partner’s reproductive health. This reflected the traditional family dynamics of 1960. However, this would soon change on *Woman’s Hour* and in Britain more widely (Laura King 2016).

Another eventual change would be *Woman’s Hour*’s engagement with the response letters to “childbirth depression” features. If *Woman’s Hour* received hundreds of letters per week, and if “childbirth” features proved immensely popular (Lloyd 2020, 120–1; Murphy 2022, 211), listeners definitely responded to Barton’s lecture. However, like most anonymous and taboo “confession” letters, *Woman’s Hour* did not broadcast any if received (Lloyd 2020, 131–3). To encourage “rational” discussions of social problems, every *Woman’s Hour* episode included 3–5 minutes of reading and answering listeners’ letters; however, Collins did not want their “personal points” on air (Lloyd 2020, 107, 111). Therefore, maternal mental illness testimonies were likely “inappropriately” personal in 1960. Against Collins’s wishes, *Woman’s Hour*’s women producers carefully shared testimonies on other taboo topics in the early 1950s, such as disability, miscarriages, and cancer, and even taboo testimonies by the late 1950s, including prostitution, birth control and pornography (Murphy 2022, 210–1). Yet, in 1960, *Woman’s Hour* did not broadcast— or perhaps no one was ready to share—personal experiences of childbirth depression.

## “Puerperal depression” on *Woman’s Hour* (July 1974)

*Woman’s Hour’s* next feature on “puerperal depression” was transmitted fourteen years later on Thursday, July 11 1974 (*Woman’s Hour* 1974). The episode aired during *Woman’s Hour*’s typical hour, 1.45–2.45pm, reaching housewives, men, and now more women at work. The social backgrounds of listeners had also changed. In 1967, the Light Programme channel was terminated and replaced by the “low-brow” channel Radio 2 (1967-present), where *Woman’s Hour* was newly broadcasted. However, in 1972, the BBC moved *Woman’s Hour* to the “middlebrow” Radio 4 (1967-present). This diversified *Woman’s* Hour’s listeners from primarily working to middle class (Murphy 2022, 212).

*Woman’s Hour*’s revived interest in “puerperal depression” was prompted by Nemone Lethbridge’s auto-fictional television play *Baby Blues* (BBC1 1973). In Lethbridge’s play, a woman experiences postnatal depression and psychosis after delivering her first baby. She eventually kills herself and her child to escape their suffering. Approximately million people watched *Baby Blues* (BBC 1974). *Woman’s Hour*, again, dedicated a lengthy eleven minutes to their 1974 feature; several minutes longer than the others, highlighting its importance. Again, up to four million people listened to the episode (Macgregor 2003, 150).

In contrast to 1960, the 1974 broadcast primarily featured women, including the producer (Mary Redcliffe), compère (Sue MacGregor), interviewer (Ann Heyno), interviewees (Ingrid Thomas, Nemone Lethbridge and an anonymous woman), a gynaecologist (Dr Katharina Dalton) and very briefly, an unnamed male psychiatrist. Clearly, women could publicly discuss puerperal depression, unlike in 1960.

Historian Tracey Loughran (2020, 135–7) argues that the print press trialled more “daring[ly]” direct and emotional testimonial styles from 1960 to 1980 to compete with the rise in television and, therefore, decline in print press audiences. I argue radio did the same to retain listeners’ attention. For instance, by the early 1970s, *Woman’s Hour* wanted their producers and presenters to be emotionally connected or invested in their topics. Consequently, *Woman’s Hour* supervisor Stephen Bonarjee deliberately appointed people with direct motherhood experience to the production team when he noticed that very few had children (Murray 2003). In turn, *Woman’s Hour*’s appointment of Redcliffe increased the number of sensitive features on motherhood, and she likely produced this particular feature because, along with her professional capability, she was a mother of three (Macgregor 2003, 165–6). Regarding the compère, MacGregor’s father was a doctor, which influenced her interest in *Woman’s Hour*’s health topics (Sue Macgregor 1998). Heyno, the interviewer, came from a school counselling background before becoming a presenter in the 1970s (For Schools, Colleges 1978). She was professionally equipped for mental health features.

As I argue in another article (2024), Lethbridge—who came from an aristocratic background—was a key spokesperson on maternal mental illness because of her *Baby Blues* play. Lethbridge accepted *Woman’s Hour*’s interview invitation because she wanted to create a safer public space for other sufferers to share their experiences. The two other mothers on *Woman’s Hour* were “ordinary” women. *Woman’s Hour*’s prioritising of “ordinary sufferers” reflected a broader cultural change in BBC radio. Previously, in the 1950s, Winnicott’s medical lectures on “The Ordinary Devoted Mother …” on *Woman’s Hour* were intentionally written for “ordinary” people to understand (Karpf 2014). However, in the early 1970s, the Radio 4 controller, Tony Whitby, went a step further. He wanted ordinary people to share their “intellectually demanding or disturbing … [and] scurrilous or comforting” experiences (David Hendy 2010). Consequently, Radio 4 encouraged more informal broadcasting styles and sensitive discussions (Lloyd 2020, 99). This reflected the growing importance of “ordinary” people’s voices and experiences in all media types by the 1970s; ordinary “ideal citizens” could now be “expert citizens[s]” (Claire Langhamer 2018, 189–90; Loughran 2020, 142–3). However, these “ideal citizens” were white “British,” heterosexual, and working-to-middle-class people (Langhamer 2018, 183–8). For example, the suffering mother on *Woman’s Hour*, Ingrid Thomas, was likely white and certainly middle-class, married and well-spoken. This illustrated the BBC’s ongoing bias against “unsuitable” working-class accents on air (Brenda Maddox 2002). Nonetheless, Thomas was one of the first “ordinary” women to share her experience of puerperal depression on twentieth-century radio; fifty years after radio had been invented. Aside from the interviewer, Thomas was given the most time to speak, further illustrating her importance.

The medic, Dr Katharina Dalton, was introduced as a “gynaecologist.” Dalton (R Greene and Katharina Dalton 1953) pioneered progesterone hormone replacement therapy in the 1950s to treat “premenstrual syndrome” (PMS). Dalton later used it to treat “puerperal melancholia” and “puerperal depression,” building on the research of gynaecologist Dr Joan Malleson (1953). Dalton was a mutual supporter of Lethbridge. They both advocated the sharing of women’s experiences to improve maternal mental illness and challenged medics who dismissed women’s health issues (Creed 2024). Finally, a male psychiatrist was perhaps invited to support and balance Dalton’s perspective on *Woman’s Hour*’s educational health feature. Yet, his anonymity and brief inclusion towards the end were perhaps deliberate to emphasise the women’s voices. Moreover, *Woman’s Hour*’s inclusion of a man could have been to stop male listeners from feeling alienated and demonstrate that puerperal depression was also their concern, not just women’s.

## The interview: experiences, variations and treatment (July 1974)

*Woman’s Hour* pre-recorded the interview on May 24 1974, airing it seven weeks later on July 11. MacGregor introduced the feature, yet unlike the 1960 equivalent, the interview began and ended with the sufferer. The points of the transcript imitated those of Barton’s 1960 lecture. However, the experiences were explained by a sufferer, not a doctor. This reflected the growing deployment of the user’s voice, rather than professional psychiatrists, to improve people’s awareness and understanding of mental illness.

For instance, Thomas began the interview by dispelling the romanticisation of “easy” and “happy” motherhood that women’s magazines and her friends had fostered. She explained how, in reality, she felt exhausted, frightened and called everything into question, including her marriage, future, and family. Thomas wanted to forget about her family and be alone. Like Barton, Heyno explained how Thomas’ experience was normal and how awareness stopped this unnecessary guilt.

Dalton then strengthened the message that “baby blues” and “puerperal depression” were common; eight in every ten mothers suffered, and one in every ten required treatment. Dalton unpacked her 1971 study based on 500 hundred women, revealing how the most “maternal” mothers—defined as women who eagerly wanted to develop a close relationship with their child—more likely developed puerperal depression because they felt more anxious throughout pregnancy and after childbirth (Dalton 1971). Thomas reiterated how this reflected her own experience; she did not expect to feel “resentment” towards the baby. Thomas also explained how she transitioned from being an “organised and … capable efficient person,” coping easily with work and domestic labour, to not coping with her husband, baby, or herself. When Dalton explained that all doctors now recognised and could treat the illness if mothers asked for help, Thomas was again presented as a brave role model for listeners. Even though medical endorsement from Dalton was important, Thomas was the heart of the feature.

Although Thomas had support—and “privilege”—from a daily cleaning lady, a helpful mother, and a very understanding husband, Thomas could not care for her baby despite believing it was the “most normal thing a woman can do.” Clearly, motherhood was still women’s “central” role in 1970s Britain.

In contrast to Barton’s lecture, *Woman’s Hour* invited a woman to share her bleak “tragedy” of puerperal depression. In graphic detail, she explained how, after a build-up of tensions, she experienced a fit of rage and committed infanticide. The woman contacted Lethbridge after watching her play. Although infanticide was not a new phenomenon (Marland 2004, 167–200), this testimony was a first of its kind and marked a milestone in women sharing infanticide experiences on the radio, albeit anonymously, for her protection and to avoid shame.

Next, like Barton, the male psychiatrist clarified the differences between “childbirth blues,” “puerperal depression” and “puerperal psychosis.” His explanations supported Dalton’s theory that maternal mental illness was hormone-related by explaining how the blues were typically over within two weeks, whereas puerperal depression tended to persist until the mother finished breastfeeding or her periods became regular again. The symptoms of puerperal psychosis, such as confusion, distorted thoughts, and a personality change, could, however, persist for life. The man advised twenty-fourhours-a-day supervision when sufferers were with their children. Dalton then promoted her progesterone replacement therapy for women’s post-childbirth “hormone imbalance;” however, Heyno explained that Thomas was treated by anti-depressants, more typically prescribed by NHS doctors. *Woman’s Hour* did not want to challenge the NHS’s orthodox treatment (Karpf 1988; Macgregor 1998).

In the penultimate section, Lethbridge read letters from sufferers who had watched her play, which contained stories similar to the aforementioned anonymous woman. Lethbridge concluded that most people wrote in to ask how they could support other women. Heyno then introduced Lethbridge’s self-help group, Depressive Anonymous, which provided telephone services and group meetings for sufferers. Like Barton’s 1960 lecture, Thomas ended the feature with positive information on how to recover. She concluded that thousands of sufferers will recover through “time … understanding … [and] rest.” Additionally, the support behind a woman’s postpartum recovery had moved beyond “sympathetic friends, nurses or doctors” to include a new grassroots organisation.

Responding to this feature, people sent hundreds of letters to *Woman’s Hour*, Lethbridge’s personal post-box, Depressives Anonymous and *Spare Rib* (Jean Gardiner 1974). These letters were not kept in the archive or read on *Woman’s Hour*. Yet, as the next case study will demonstrate, from the mid-1970s, a watershed of puerperal depression testimonies—instigated by everyday people—emerged in the press, radio, television, and non-medical books for the first time in Britain, persisting into the future (Creed 2024, 5, 18). This *Woman’s Hour* feature was part of this culture change, contributing to open discussions of maternal mental illness.

## “Postnatal depression” on *Woman’s Hour* (June 1985)

Eleven years later, on Thursday, June 13 1985, between 2 and 3 pm, the topic again featured on *Woman’s Hour*, but this time termed “postnatal depression.” In the 1970s, this term gradually replaced “puerperal depression.” The 1985 feature appeared after the watershed of BBC media and self-help literature on postnatal depression, snowballing from the mid-1970s (Creed 2024, 5, 18). Consequently, the discussion was more of an update and considerably less taboo. It echoed the print press and television by providing more personal accounts rather than a ground-breaking or controversial broadcast. For more “radical” interviews on postnatal depression, people would look to *Spare Rib* (1972-–94), an influential feminist magazine.

Moreover, the title, “postnatal depression and hormone therapy treatment,” illustrated a focus on unorthodox treatment, more widely accepted in the 1970s. This was, in part, reflective of the Woman’s Liberation Movement’s (WLM) criticisms of doctors’ liberal prescribing of pharmacological treatments, including anti-depressants and tranquilisers, which feminists believed “sedate[d]” and helped women “cope” with sexism, rather than offering “solutions” (Women in Mind 1986). Britain’s WLM emerged in 1968 but was most active in the 1970s and 1980s, tracing the lifespan of Britain’s most high-profile feminist magazine, *Spare Rib*. The WLM campaigned for women’s political, economic, and social equality through consciousness-raising media, often emotionally charged and angry. *Woman’s Hour*, however, needed to appear more neutral to Britain’s medical and media institutions because it was managed by the BBC, which supported the NHS. Consequently, *Woman’s Hour*’s production team were wary of challenging the NHS and supporting feminist movements (Macgregor 1998). This was evidenced by MacGregor’s strike-out of a woman’s angry testimony from the script, which explained how her GP had prescribed pharmacological drugs for postnatal depression, telling her to “get on with it.” Feminist media—including *Spare Rib, Trouble & Strife, WIRES* and *Outwrite*—encouraged angry testimonies, but not *Woman’s Hour*.

Nonetheless, *Woman’s Hour*’s production team presented a thorough “puerperal depression” discussion, dedicating thirteen minutes to the feature, the longest of six other reports. It was, however, the only feature on motherhood, whereas the others explored women’s careers. *Woman’s Hour* focused on women’s paid labour to balance the motherhood feature, which now exasperated many listeners in the 1980s. Again, all the speakers were women except for one man. MacGregor was still the compère. The producer, Judy Swallow, was not mentioned in *Woman’s Hour*’s history. Finally, the interviewer, Sue Margolis, had joined *Woman’s Hour*’s London studio in the early 1980s.

The media trend that prioritised women’s expertise over medics had intensified; *Woman’s Hour* encouraged three “ordinary” postnatal depression sufferers—Denise, Irene and Margaret—to speak the most. The three women were anonymous to listeners, and the BBC Archive did not keep personal information of their class, age, ethnicity, race or location. This, again, offered protection and suggests stigma was still somewhat widespread, albeit much less when compared to the 1960 lecture when testimonies were impermissible.

All three women in the 1985 feature were, again, in heterosexual marriages because single or lesbian mothers elicited more taboo and less sympathy from listeners (Rebecca Jennings 2023; Pat Thane and Tanya Evans 2012). However, one of the husbands spoke for the first time and shared a family experience of postnatal depression. This contrasted greatly with the 1960 lecture, where Barton’s advice for husbands was redacted.

By the 1980s, the BBC often instructed husbands to help with childcare, support their partner’s mental health, and take note of the dynamic between their wife and children (*The Man Alive Report* 1978). *Woman’s Hour* listeners were delighted by this change as they found “wife and husband teams … inspiration[al]” (*Woman’s Hour* 1985c, Letter 1). Beyond radio, this cultural change emerged on BBC, ITV and Channel 4 television (*Post-Natal Depression Who Cares?* 1984; *Lady Sings the Blues*; 1985), reflecting Britain in general.

The leading medic was, again, Dalton. Dalton was now a household name for testifying in high-profile criminal cases about the effects of PMS on women’s state of mind. Dalton promoted postpartum hormone replacement therapy on television in 1978, endorsed by television personality and former postnatal depression sufferer Esther Rantzen (*The Man Alive Report*). Two years later, Dalton published her fifth book, *Depression After Childbirth* (1980), which included a foreword by Rantzen. Already a quasi-celebrity, Dalton’s partnership with Rantzen helped her unorthodox therapy gain further recognition. As a leading “endocrinologists” working on postnatal depression, Dalton was an ideal medical spokesperson. The mainstream British press, unlike the North American press (Rachel Louise Moran 2024), strongly supported Dalton, especially the BBC.

The man in the feature, Dr Ian Simpson, was a GP from Reading, which reflected a new crucial layer of medical expertise. In the 1980s, psychiatric services were increasingly underfunded, and the role of GPs broadened (John Mohan 1995, 242). Subsequently, GPs were more likely to provide referrals for postnatal depression. To maintain editorial balance, Simpson supported Dalton while sympathising with other GPs and psychiatrists, whom Dalton, the three women and one husband challenged.

## The interview: experiences, causes and treatment

Like the 1974 feature, the interview focused on postnatal depression testimonies, causes and treatments; it merely revisited the topic. MacGregor began by asserting, “1 in 10 women suffer feelings of depression, inadequacy and helplessness” within the first year of having a baby. She then reported that the standard treatments were tranquilisers and anti-depressants, mimicking *Woman Hour*’s 1960 and 1974 reports. However, Dalton maintained that postnatal depression was a “physical” illness because her treatment, via a natural form of the hormone progesterone, had been successful for over forty years. The three former sufferers claimed that Dalton’s hormone therapy treatment cured them.

Building on previous features, the second section explained how all three women experienced postnatal depression differently. The first speaker felt “extreme panic” and could not eat, sleep, concentrate or “switch off.” The second speaker cried most of the day and felt tense, tired, anxious, and miserable. In contrast, the third woman felt “excited,” “high” and became “destructive” and “out of character;” the nurses and doctors had to pin her down to inject sedatives. She also developed paranoia, thinking the nurses were harming her baby and others on the ward. To extend the long “list” of wide-ranging symptoms, Margolis added that other mothers could develop “lethargy,” “aggression,” and “inexplicable pains and palpitations,” alongside severe psychotic symptoms of “delusions and hallucinations.” Clearly, medics now commonly categorised a wider variety of symptoms as postnatal depression.

In previous features, the discussion on causation was speculative, but in this episode, it formed more of a debate. Margolis explained that doctors attributed “tiredness, a change in lifestyle, loneliness and boredom;” however, Dalton disagreed, defending that mothers who adopt babies at birth experienced these changes but did not become clinically depressed. She, therefore, attributed postnatal depression to hormonal changes following childbirth, mainly the loss of progesterone after nine months of abundance. Dalton reassured listeners that progesterone supplements had no health risks because she had trialled the natural hormone for forty years.

The fourth section informed listeners on when postnatal depression developed and how long it lasted. Dalton explained that it occurred between the first day and six months after giving birth or after a woman’s first menstruation, when she stopped breastfeeding, or started the contraceptive pill. Dalton explained that it could last any length of time, in contrast to Barton’s optimistic lecture that all illnesses were curable.

Moreover, by the mid-1980s, people more openly shared the shocking consequences of untreated postnatal depression. This reflected a broader shift in 1980s television and print press news; Britain became more accepting of hard-hitting visuals and rhetoric to influence people’s actions if it aimed to improve people’s health (Jane Hand 2020). On *Woman’s Hour*, one of the three speakers, Irene, shared her devastating experience of infanticide and multiple suicide attempts to demonstrate the consequence if sympathetic doctors and “successful” treatments, like hormone replacement therapy, were inaccessible. Irene’s GP was unsympathetic; the psychiatrist briefly visited her, and treatments like tranquilisers and anti-depressants proved ineffective. She recovered using hormone replacement therapy, but only after the “tragedy.” The other speaker, Denise, also shared how her unsympathetic GP prescribed a tranquiliser, Ativan, which did not cure her. Simpson, a GP himself, agreed that anti-depressants and tranquilisers were “inappropriate” because they increased people’s confusion and lethargy. He instead supported Dalton’s theory and therapy of natural progesterone. Margolis confirmed that Simpson was not alone in this opinion; however, the GPs who prescribed progesterone were “few and far between;” most “wr[o]t[e] off” suffering mothers as “neurotic.”

In the next section, Margaret shared her “frighten[ing]” experience in a psychiatric hospital to reinforce how traditional treatments (tranquilisers, Largactil and ECT) were ineffective. Margaret’s husband, Richard, refused to accept the psychiatrist’s prediction that his wife required two years of recovery and read Dalton’s book on postnatal depression. Richard originally “battled” with the psychiatrist to get progesterone treatment for Margaret. Nonetheless, after five days of progesterone treatment, Margaret fully recovered and never returned to the sanatorium. The “hostile” psychiatrist refused to accept the treatment had cured Margaret, instead believing it was the drugs he prescribed. In the penultimate section, Dr Simpson defended the doctors, arguing that they were uninformed and were not “prejudice[d]” against progesterone therapy. He argued that GPs, in particular, were expected to know everything about every illness, which was impossible. Simpson maintained that hormone therapy had not been taught in medical school or written in textbooks.

The final section was more upbeat, albeit with a warning. Margaret was told that if she had another baby, she would not recover; however, both Margaret and Irene gave birth again and, following progesterone treatment, did not suffer. In contrast, Denise suffered for months until she finally accessed the treatment. Denise concluded that she had “this very strong feeling in [her] gut,” rather than “scientific proof,” that progesterone “cured” her. The listeners who felt abandoned by Britain’s dismissive medical system would have valued Denise’s “gut” feeling over the “science” behind it.

By the 1980s, *Woman’s Hour* was highly influential. Within the first week of the broadcast, presenters remarked on the “[large] number” of response letters posted from all over Britain (BBC Written Archives, Woman’s Hour 1985b, 1985c). *Woman’s Hour* shared some with their listeners. This reflected both women’s and the media’s confidence in discussing maternal mental illness. Most women cathartically echoed their own distressing experiences (1985b, Letter 1). Others supported Dalton’s hormone treatment as it had worked for them (1985b, Letter 2). One woman warned about the difference between “natural progesterone” and “progestogen,” which was a synthetic alternative given in pill form by GPs. She explained how artificial progestogen temporarily stopped her natural production of progesterone, which worsened her mental health (1985b, Letter 3).

*Woman’s Hour* also read out letters from people who were angry about the feature for different reasons. Some women were concerned about the “hormonal” explanation and treatment. They argued that it dismissed “the social and personal pressures on women to become mothers, which also contribute[d] to stress and depression” (underlined in the letter) (1985c, Letter 3). Whereas some men were frustrated by the “female selfindulgence concerning post-natal, post-caesarean and pre-menstrual problems” on *Woman’s Hour* (1985c, Letter 2). These types of letters connected to a new and reoccurring complaint about *Woman’s Hour*, or, as they ridiculed, “Mother’s Hour,” in the 1980s. Many women became exasperated by *Woman’s Hour*’s focus on motherhood, as it alienated the growing numbers of single women who could not or did not want children (1985a, Letter 1). Clearly, for younger generations, women’s “central” role as mothers was culturally weakening.

## Conclusion

As this article illustrated, *Woman’s Hour* exemplifies how mothers’ mental health, and mental health in general, became a significant post-war concern (Angela Davis 2012) and how media and medical institutions attempted to improve “puerperal depression.” People’s understanding of postnatal depression grew alongside ever-expanding variations in narratives, treatments, and public reception.

The types of people and their listening environments also expanded. When the first postnatal depression feature was transmitted in 1960, working-class housewives listened at home and men at work. By the mid-1980s, working and middle-class women and men listened at home and at work. *Woman’s Hour* also approached the originally taboo topic more comfortably. I argue that this both reflected and contributed to the destigmatisation of maternal mental illness in medical, media, feminist, and public spaces across the late twentieth century.

This article explored how *Woman’s Hour* experimented with groundbreaking mental health coverage, including “childbirth depression,” at the turn of 1960, albeit cautiously and tactically. However, when television replaced radio as the dominant form of public health communication, *Woman’s Hour* merely echoed other media on postnatal depression; radio was no longer at the forefront of de-stigmatising taboo subjects. When *Woman’s Hour* revisited the topic in 1985, they imitated the mainstream “nuclear family” press coverage; all presented controlled debates from “wives” and their “husbands.” If feminists wanted controversial, uncensored and emotionally charged debates, which fiercely challenged NHS practitioners, they instead read “radical” feminist media.

Regarding medical narratives, this article demonstrated how growing stakeholder groups debated maternal mental illness on air. When *Woman’s Hour* began broad-casting maternal health advice, they prioritised partnerships with and feared “objections” the Ministry of Health and the BMA (Lloyd 2020, 129–30), hence the anonymous, sensitive and positive medical lecture in 1960. However, by the 1980s, *Woman’s Hour* encouraged psychiatrists, gynaecologists, endocrinologists, and GPs to debate postnatal depression. Since the 2000s, *Woman’s Hour* has invited even more stakeholder groups, including politicians and NGOs (e.g., the National Childbirth Trust).

‘Regarding sufferers’ narratives and “expertise”, *Woman’s Hour* increasingly supported the sufferers who challenged NHS medics when maternity services were inadequate, even if it included testimonies of infanticide and attempted suicide. This acceptance of “hard-hitting” narratives to raise awareness, drive change, and encourage sufferers to seek NHS support was in tune with other media’s use of “shock tactics” in 1980s health campaigns. More recently, Woman’s Hour (2019) comfortably broadcasts emotionally charged narratives, even if they deviate from the proposed structure. In addition, maternal mental illness sufferers over eighteen now disclose their full names, demonstrating a decline in communal shaming. However, these narratives still overwhelmingly reflect the experiences of white “British,” middle-class, heterosexual and married women. At present, *Woman’s Hour* has not invited brown or black women or homosexual or non-binary parents to share their experiences of postnatal depression. Yet, they have addressed paternal postnatal depression twice (2016; 2018).

This article also illustrated the expansion in treatments and who became responsible for supporting sufferers. In 1960, greater accountability was placed on the sufferer alongside medics, but not husbands. By the 1980s, the responsibility expanded to all family members, friends, neighbours, and a growing range of medical, media, and government maternity care services. The medical and pharmacological treatments also expanded into “alternative” hormone therapy. Nowadays, psychological talk therapies are offered for mothers and their partners.

Finally, regarding reception, women transitioned from ambiguously demanding and not being allowed to publicly respond to a feature on “childbirth depression” to becoming exasperated by another maternity issue on “Mother’s Hour.” To conclude, women shifted from being prohibited from sharing their experiences to being able to shamelessly complain about “Mother’s Hour” topics. This, in itself, reflected motherhood’s declining status in England by the twenty-first century. Many women had started to challenge their “central” role as mothers, even though the British media, like *Woman’s Hour*, had steadily evolved to provide some mothers with better support.

## Footnotes

1 Established in 1919, the Ministry of Health was replaced by the Department of Health and Social Security in 1968.
